# Influence of epinephrine reactivity to stress on meat quality in goats

**DOI:** 10.1093/tas/txae078

**Published:** 2024-05-28

**Authors:** Arshad Shaik, Phaneendra Batchu, Aditya Naldurtiker, Priyanka Gurrapu, Brou Kouakou, Thomas H Terrill, Govind Kannan

**Affiliations:** Agricultural Research Station, Fort Valley State University, Fort Valley, Georgia 31030, USA; Agricultural Research Station, Fort Valley State University, Fort Valley, Georgia 31030, USA; Agricultural Research Station, Fort Valley State University, Fort Valley, Georgia 31030, USA; Agricultural Research Station, Fort Valley State University, Fort Valley, Georgia 31030, USA; Agricultural Research Station, Fort Valley State University, Fort Valley, Georgia 31030, USA; Agricultural Research Station, Fort Valley State University, Fort Valley, Georgia 31030, USA; Agricultural Research Station, Fort Valley State University, Fort Valley, Georgia 31030, USA

**Keywords:** epinephrine, goats, meat quality, metabolomics, stress

## Abstract

The magnitude of physiological responses to a stressor can vary among individual goats within a herd; however, whether these differences can differentially affect meat quality is not known. This study was conducted to determine the influence of the magnitude of epinephrine response (ER) to acute stress on muscle metabolome and meat quality in goats. Male Spanish goats (6 mo old) were transported for 180 min. (*N* = 75 goats; 25 goats/d) to impose stress. Blood samples were obtained after transport for analysis of physiological responses. Goats were slaughtered using humane procedures and samples were collected for muscle metabolomics and meat quality analyses. The data obtained from blood and muscle/meat analysis were then categorized based on epinephrine concentrations into low (LE), medium (ME), and high (HE) ER groups (*n* = 12/ER group). The physiological and meat quality variables were analyzed as a Completely Randomized Design in SAS, and metabolomics data were analyzed using R software. Plasma glucose concentrations were significantly high in the HE group, low in the LE group, and intermediate in the ME group (*P *< 0.05). However, leukocyte counts and cortisol, norepinephrine, blood urea nitrogen, and creatine concentrations were not different among the ER groups. Muscle (Longissimus dorsi) glycogen concentrations (15 min postmortem) were significantly higher (*P* < 0.05) in the ME and LE groups than in the HE group. However, postmortem Longissimus muscle pH and temperature (15 min and 24 h), 24 h calpastatin and desmin levels, and rib chop color (L*, a*, and b*), cooking loss, and Warner-Bratzler shear force values were unaffected by ER. Targeted metabolomics analysis of *Longissimus* muscle (15 min) revealed that diacyl phosphatidylcholines (C38:0; 40:6) and sphingomyelin (C20:2) were significantly different (*P* < 0.05) among the ER groups, with the concentrations of these metabolites being consistently high in the LE group. These differential muscle metabolite concentrations suggest that ER can influence biochemical pathways associated with cell membrane integrity and signaling. ER had a significant effect on dopamine concentrations, with the levels increasing with increasing levels of ER. The results indicate that differences in epinephrine reactivity can influence selected physiological responses and muscle metabolites; however, it does not significantly influence meat quality attributes.

## Teaser

Individual differences in stress reactivity do not significantly influence the meat quality characteristics in goats.

## Introduction

In the United States, most meat goats are produced in the southeast, with Texas leading all other US states in output (Qushim et al., 2015). These animals are transported for long durations, for example, from western Texas to farms in several other states, with some journeys lasting up to 20 h depending on the distance (Kannan et al. 2023). Goats intended for slaughter are typically transported 2 to 3 h prior to slaughter which can impose significant stress and affect the quality of meat if slaughtered without overnight holding.

Stress associated with preslaughter handling and transportation depletes muscle glycogen, resulting in a higher final pH and lower quality attributes of meat (Kadim et al., 2006; Toplu, 2014). The development of meat quality is dependent upon the extent of postmortem muscle metabolism, which in turn is influenced largely by the intensity and duration of antemortem stress. Goat carcasses have final pH values between 5.8 and 6.2, an indication that goats are likely stressed prior to slaughter (Simela et al., 2004).

Research on other livestock species has shown that reactivity to stress can be influenced by genetics. For example, cattle of different breeds exhibited different stress responsiveness as shown by urinary catecholamine levels (Muchenje et al., 2009). These authors found a relationship between elevated catecholamine and darker meat and further observed that the relationship between stress reactivity and meat quality characteristics may be different among the different cattle breeds. Besides genetics, stress reactivity can be influenced by the environment and whether or not the animals have experienced human handling previously (Terlouw, 2015). The author observed that stress reactivity is also different among individual animals with similar genetic makeup and raised and slaughtered under similar conditions, which is reflected in the differences in the extent of postmortem muscle pH decline (Terlouw, 2015). Studies in pigs have shown that the incidences of fighting and the resultant carcass damage were associated with elevated physiological stress responses, including plasma epinephrine concentrations, which in turn resulted in glycogen depletion and higher ultimate pH of meat (Fernandez et al., 1994; Foury et al., 2011). Emotional reactivity and physiological stress reactivity are consistent over time in individual animals both in the wild and in captivity (Carere et al., 2010). Such individual differences exist among goats within a herd in the magnitude of physiological response to a stress stimulus, probably due to differences in personalities, although there is very little information available on this aspect in goats.

Researchers have used several physiological indicators to assess stress in goats. Kadim et al. (2006) observed increased levels of plasma cortisol, epinephrine, norepinephrine, and dopamine in goats subjected to transportation stress. In addition, blood glucose and urea nitrogen concentrations and creatine kinase activity also increase due to transportation stress in horned meat goats (Kannan et al., 2000). Physiological stress or physical activity depletes muscle glycogen reserves, which may result in higher final meat pH, darker meat color, and tougher meat (Chulayo et al., 2012). Acute stress-induced antemortem depletion of muscle glycogen is a direct effect of catecholamine release, primarily epinephrine, and the consequent increase in glycogenolysis (Kannan et al. 2003). In addition to the traditionally used objective and subjective methods of evaluating meat quality, meat metabolomic profiling has been used in recent studies to identify biomarkers of meat quality traits, such as marbling and tenderness (Jeong et al., 2020; Lee et al., 2022).

Both animal welfare and meat quality are negatively impacted by severe preslaughter stress (Kannan et al., 2000), although not all animals respond to a stressor to a similar degree, likely because of differences in basal secretion, how they perceive and react to a stressor, or both (Mapiye et al., 2011). To what extent these individual differences in stress reactivity influence meat quality is not fully understood in goats. Epinephrine concentration is considered a good indicator of stress response, and its release directly influences the extent of muscle glycogen breakdown, postmortem glycolysis, and pH decline. Therefore, categorizing goats based on their epinephrine reactivity to acute stress is a logical approach to evaluate the effect of response rate on meat quality in goats.

The null hypothesis tested was that the meat quality characteristics in goats with different stress reactivity are similar. The objective of this study was to determine the muscle metabolomic profiles and meat quality characteristics in goats as affected by the magnitude of epinephrine response (ER) to acute stress.

## Materials and Methods

### Animals

The animal care protocol for this study was reviewed by the Fort Valley State University (FVSU) Agricultural and Laboratory Animal Care and Use Committee following the ADSA-ASAS-PSA Guide for Care and Use of Agricultural Animals in Research and Teaching (Ag Guide, 2020) and approved (Approval Number: F-R-02-2020). The experimental animals were uncastrated male Spanish goats (6 mo old; BW = 29.7 ± 2.03 kg), maintained as part of a larger herd in a paddock with natural range forage, predominantly comprised of Bermudagrass (*Cynodon dactylon*) and a concentrate pellet supplement (18% crude protein, 4% crude fat, 15% crude fiber). The goats also had access to hay, water, and shelter at all times.

For this experiment, a total of 75 goats were slaughtered in three replicates (25 goats/d). For each replicate, the animals were taken to the processing facility the day before slaughter, weighed, and kept in holding pens overnight with no feed. On the day of slaughter, the goats were transported for 180 min to impose stress using a livestock trailer (5.3 × 2.3 m) that allowed a floor space of 0.49 m^2^/goat (Supplementary Figure 1). The all-aluminum trailer (Featherlite, Model 8107, Featherlite Trailers, Cresco, IA, USA) had a skid-resistant extruded aluminum floor. A ramp was not used for loading and unloading goats since the trailer floor was only 32 cm from the ground level. On each processing day, transportation started at 0600 hours and ended at 0900 hours. The average temperature on the days of transportation was 15.5°C, and the average relative humidity was 77.0%. Blood samples were collected after transportation, and the goats were weighed and then slaughtered using standard humane procedures.

Blood samples were used to determine differential leukocyte counts and glucose, urea nitrogen (BUN), creatine, epinephrine, norepinephrine, and cortisol concentrations, in addition to a targeted metabolomics analysis. Carcass and meat quality characteristics assessed were hot and cold carcass weights, meat pH and temperature, glycogen, color, shear force values, cooking loss, calpastatin abundance, and desmin. Based on the ER levels (ER), all physiological and meat quality data were categorized into three groups: low (LE, < 29.9 ng/mL), medium (ME, 30.0 to 59.9 ng/mL), and high (HE, > 60.0 ng/mL) ER groups. These cutoff levels were primarily derived from data distribution; epinephrine concentrations in the current study ranged from 10.1 to 97.3 ng/mL. In addition, epinephrine concentrations observed for Spanish goats in previous studies were used as guidance. The average levels in goats ranged from 7.0 ng/mL when not subjected to any stress treatment and held in home paddocks (Kannan et al., 2021) to 34.8 ng/mL when held in slaughter plant holding pens (Naldurtiker et al., 2022). The latter study also revealed a 90-min transportation of Spanish goats results in an average epinephrine concentration of 91.1 ng/mL. While these studies provided some direction in establishing the LE and HE levels, the ME limits were set arbitrarily such that the range was between LE and HE with the required number of animals. To include the same number of animals in each group, data from only 36 experimental units that fell within one of the three established ER ranges were used in the statistical analysis (12 animals/ER group). Assuming that an animal’s personality and its alarm reaction to stress may be consistent throughout its life, live weights were also recorded.

### Blood Sampling

Blood samples were collected from the jugular vein by trained personnel immediately before and after transportation prior to slaughter using disposable needles and vacutainer tubes (Becton, Dickinson and Company, New Jersey) containing 81 µL of 15% EDTA solution. Blood samples collected were stored on ice before being centrifuged (Sorvall Lynx 6000, Thermo Electron LED GmbH, Germany) at 1,000 × g for 10 min for separation of plasma. Blood samples were also collected separately in 3 mL vacutainer tubes coated with EDTA (K3) for hematology analysis.

### Differential Leukocyte Counts, Glucose, BUN, and Creatine

For glucose, BUN, and creatine, blood samples were collected separately in 3 mL vacutainer tubes coated with EDTA (K3). These blood metabolites and differential leukocyte (neutrophil, lymphocyte, monocyte, and eosinophil) counts were determined using a VETSCAN HMS Hematology Analyzer (Abaxis, Union City, CA) according to the manufacturer’s protocol.

### Cortisol

The Cortisol ELISA (enzyme-linked immunosorbent assay) Kit (Abnova, Taipei, Taiwan) was used to measure plasma cortisol concentrations according to the procedure described by Kannan et al. (2021). Briefly, a plasma sample containing an unknown amount of cortisol was measured (unlabeled antigen) and mixed with a standard amount of the same substance’s labeled derivative (labeled antigen). The labeled and unlabeled antigens were then allowed to compete for high-affinity binding sites on the plate’s limited number of antibodies. The amount of labeled antigen in the sample is reversibly proportional to the concentration of unlabeled antigen after the free antigen is washed away. Actual concentrations in unknown samples were determined using a standard curve based on known concentrations of unlabeled antigens analyzed concurrently with the unknowns. An enzyme label is used in this kit. During the 1 h incubation period, the biospecific reaction occurs. After washing, a substrate solution was added, and the enzyme was allowed to react for a set amount of time before the reaction was stopped. To summarize, goat plasma samples (25 μL) were used to coat wells of 96-well microplates before adding the cortisol enzyme conjugate solution (100 μL per sample). The plates were incubated at 37 °C for 1 h before being washed four times with the wash solution, and the 3,3ʹ,5,5ʹ-tetramethylbenzidine (TMB) color reagent (100 μL) was added to each well. The reaction was stopped by adding 50 μL of the stop solution, and optical density was measured with a microwell reader at 450 nm (Synergy HTX Microplate Reader, Bio-Tek, Winooski, VT). Cortisol concentrations were determined against a standard curve created using the standards and according to the manufacturer’s instructions. The minimum detectable concentration of cortisol by this method is 1.0 ng/mL.

### Catecholamines

The Epinephrine/Norepinephrine ELISA Kit (Abnova, Taipei, Taiwan) was used to determine plasma epinephrine and norepinephrine concentrations (Kannan et al., 2021). Using a cis-diol-specific affinity gel, epinephrine and norepinephrine were extracted, acylated, and then converted enzymatically. The antigen was bound to the microtiter plate’s solid phase. The derivatized standards, controls, and samples, as well as the solid phase bound analytes, were given a fixed number of antibody binding sites to compete for. Free antigen and antigen-antibody complexes were removed by washing after the system reached equilibrium. Using TMB as a substrate, the antibodies bound to the solid phase were detected by an anti-rabbit IgG-peroxidase conjugate, and the reaction was monitored at 450 nm. The microtiter plates were read for absorbance values using a Synergy HTX Microplate Reader (Bio-Tek, Winooski, VT). The limits of detection by this method were 0 and 200 ng/mL for epinephrine and norepinephrine, respectively.

### Carcass Processing and Muscle/Meat Sampling

The goats were slaughtered and processed according to standard humane protocols at 0930 hours on each day of the trial. Goats were stunned using a captive-bolt pistol by a trained individual immediately before exsanguination. Following slaughter, two samples were collected (15 min postmortem) from the *Longissimus lumborum* muscle on the right side of the hanging carcass for glycogen and targeted metabolomics analyses. Muscle samples were wrapped in aluminum foil and quickly frozen in liquid nitrogen before being transferred to a −80 °C freezer. The carcasses were weighed and cooled at 2 °C for 24 h. Each carcass was then weighed and fabricated after 24 h of storage, and 2.5 cm-thick rib chops (*Longissimus thoracis*) were collected from each side of the carcass. Four chops from the right side were allowed to bloom for 40 min, and a Miniscan XE Plus calorimeter was used for the determination of color values (L* a* b*; HunterLab, Reston, VA). Four chops from the left side were vacuum packed and frozen at −20 °C and were later (< 1 mo) used to determine Warner-Bratzler shear force (WBSF) values and cooking loss percentages. One additional rib chop from each side was designated for calpastatin and desmin analysis. Longissimus muscle samples were dissected from the chops, quick-frozen in liquid nitrogen, and stored at −80 °C until analyzed.

### Muscle pH and Temperature

Using a portable combination pH meter with a penetrating probe (Pakton® Model OKPH1000N, Vernon Hills, IL), initial pH and temperature were recorded immediately (15 min postmortem) after skinning and evisceration and after 24 h of slaughter (final pH) on the left side of the hanging carcass in the loin region. To measure pH and temperature, the probes were inserted directly into the *Longissimus lumborum* muscle, and the values were recorded.

### Muscle Glycogen

Glycogen concentrations in muscle were determined using a Glycogen Assay Kit (Abnova Corporation, Tapei, Taiwan) as described by Naldurtiker et al. (2022). In this assay, the glucoamylase enzyme hydrolyzes glycogen to glucose, which is then specifically oxidized to produce a product that reacts with an OxiRed probe to produce a color that is measured calorimetrically. A total of 10 mg of tissue was taken per sample, homogenized in 200 µL of distilled water, and boiled for 10 min to inactivate enzymes. The boiled samples were centrifuged for 10 min at 18,000 × g to remove insoluble material before collecting the supernatant. The supernatant (10 µL) was collected, and the final volume was adjusted to 50 µL/well with a hydrolysis buffer. The plates were then incubated at room temperature for 30 min. Following incubation, 50 µL of the reaction mixture was added to each well and plates were incubated for another 30 min at room temperature. Finally, using a Synergy HTX Microplate Reader, the absorbance was measured at 570 nm (Bio-Tek, Winooski, VT).

### Cooking Loss

Cooking loss was determined according to the procedure described by Kannan et al. (2002). The chops were thawed at 4 °C before being placed on aluminum pans and covered with aluminum foil. Prior to cooking, the samples were weighed. The chops were cooked to an internal temperature of 71 °C in a convection oven (Maytag model, Redwood, CA). Using thermocouple thermometers, the internal temperature of a representative cut from each pan was determined (Fisher Scientific, Suwanee, GA). The thermocouple probe was placed in the geometric center of the muscle in a randomly selected cut from each pan. The samples were then allowed to come to room temperature before being weighed to determine the percentage of cooking loss.

### Warner-Bratzler Shear Force

Cooked samples were wrapped in aluminum foil and cooled overnight at 4 °C. Cuts were removed from the refrigerator and placed on a countertop for 2 h to allow them to come to room temperature. From each chop, a minimum of two 1-cm diameter cores were taken parallel to muscle fiber orientation (Kannan et al., 2002). The WBSF values were determined using a TA-XT2 Texture Analyzer (Texture Technologies Corp., Scarsdale, NY), with samples sheared at right angles to muscle fiber orientation using the Warner-Bratzler shear attachment (Texture Technologies Corp., Scarsdale, NY). The texture analyzer was configured with a 5-kg load cell and a crosshead speed of 200 mm/min.

### Calpastatin Abundance

The concentration of calpastatin (CAST) in muscle was determined using a CAST ELISA kit (MyBioSource, San Diego, CA) according to the procedure used by Naldurtiker et al. (2022). The competitive enzyme immunoassay technique was used in the CAST ELISA kit, which includes a polyclonal anti-CAST antibody and a calpastatin- horseradish peroxidase (CAST- HRP) conjugate. In a pre-coated plate, the assay sample and buffer were incubated for 1 h with a CAST-HRP conjugate. The wells were decanted and washed five times after incubation. The wells were then incubated with HRP enzyme substrate. The result of the enzyme-substrate reaction is a blue complex. Finally, a stop solution was added to halt the reaction, which caused the solution to turn yellow. A Synergy HTX Microplate Reader was used to measure color intensity spectrophotometrically at 450 nm (Bio-Tek, Winooski, VT). Because CAST from samples and the CAST-HRP conjugate compete for the anti-CAST antibody binding site, the color intensity is inversely proportional to the CAST concentration. Because the number of sites is limited, as more CAST from the samples occupy them, fewer sites are left to bind the CAST-HRP conjugate. A standard curve was created to show the relationship between the optical density values and the concentrations of standards. This standard curve was used to interpolate the CAST concentration in each sample.

### Desmin

The concentration of desmin (DES) in muscle was determined using a DES ELISA kit (MyBioSource, San Diego, CA) as described by Naldurtiker et al. (2022). A competitive enzyme immunoassay technique was used in the DES ELISA kit, which includes a polyclonal anti-DES antibody and a desmin-horseradish peroxidase (DES-HRP) conjugate. In a pre-coated plate, the assay sample and buffer were incubated for 1 h with the DES-HRP conjugate. The wells were decanted and washed five times after incubation. The wells were then incubated with HRP enzyme substrate, and the enzyme-substrate reaction resulted in a blue complex. A stop solution was then added to halt the reaction, which caused the solution to turn yellow. The color intensity was measured spectrophotometrically at 450 nm using a Synergy HTX Microplate Reader (Bio-Tek, Winooski, VT). Because DES from samples and DES-HRP conjugate compete for the anti-DES antibody binding site, the color intensity is inversely proportional to the DES concentration. Because the number of sites is limited, as more DES from the sample occupies the sites, fewer sites are left for the DES-HRP conjugate to bind. A standard curve was created to interpolate the DES concentration in each sample.

### Muscle Metabolomics

Samples from the *Longissimus lumborum* muscle of goat carcasses collected at 15 min postmortem were delivered to The Metabolomics Innovation Center (TMIC) at the University of Alberta (Edmonton, Alberta, Canada) for targeted metabolomics analysis. Using a combination of direct injection mass spectrometry and a reverse-phase LC-MS/MS custom assay, a targeted quantitative metabolomics method was utilized to analyze the samples. This custom assay was used in combination with a mass spectrometer to identify and quantify up to 150 different endogenous metabolites, including amino acids, acylcarnitines, biogenic amines and derivatives, uremic toxins, glycerophospholipids, sphingolipids, and sugars. In this approach, derivatization and extraction of analytes were combined with selective mass spectrometric detection using multiple reaction monitoring pairs. An ABSciex 4000 Qtrap tandem mass spectrometry instrument (Applied Biosystems/MDS Analytical Technologies, Foster City, CA) with an Agilent 1260 series UHPLC system (Agilent Technologies, Palo Alto, CA) was used for mass spectrometric analysis. An LC approach was used to deliver the samples to the mass spectrometer, followed by a direct injection method. Analyst 1.6.2 was used to analyze the data.

### Statistical Analysis

The data were analyzed as a Completely Randomized Design in SAS. Levene’s test and Shapiro–Wilk’s test were used to check for homogeneity of variances and normality of data, respectively. Blood metabolite and hormone concentration data were transformed to a log scale to meet the assumptions of ANOVA when required. When significant by ANOVA, the means were separated using the *P*-diff procedure. Pearson correlation analysis was also conducted to study the association between selected variables and ER.

Metabolomics data were analyzed by TMIC. Data from all 36 muscle sample extracts were used for metabolomics analysis. The metabolites with more than 20% missing concentrations and those with identical concentrations for all samples (ex. 0 μM) were deleted from the datasets. One-way ANOVA was conducted using the Kruskal–Wallis test since the data for all groups were not normally distributed and post hoc tests were conducted using Dunn’s test with Benjamini Hochberg False Discovery Rate correction for multiple comparisons. The Cliff’s Delta method (Vargha and Delaney, 2000; Macbeth et al., 2011) was used to calculate the effect size, and the ratio between group medians was used to determine the fold change.

Principal component analysis and partial least square discriminant analysis (PLS-DA) were performed using Metaboanalyst R. The models were tested for performance and the absence of overtraining with 10-fold cross-validation. A permutation test was conducted to assess the statistical significance of the PLS-DA model. A model was considered statistically significant if *P* < 0.05. Using variable importance in projection (VIP) scores, the metabolites were then plotted according to their importance in separating the different ER groups based on the PLS-DA results. The metabolite was considered significantly involved in the separation of the ER groups when a VIP score was > 1.5.

Concentration ranges for each metabolite were calculated to generate a heatmap. The normalized values (0 to 1) were used to create the heatmap.

## Results

### Physiological Indicators of Stress in Goats

Plasma glucose concentrations were higher in the HE, low in LE, and intermediate in the ME group (*P* = 0.0383, Table 1). Plasma cortisol and norepinephrine concentrations, plasma BUN and creatine concentrations, and neutrophil and lymphocyte counts were not affected by ER grouping.

**Table 1.** Effects of differences in epinephrine response to acute stress on certain other physiological indicators of stress in goats

Variable	Epinephrine response level			SEM	*n*	*P*-value
	LE	ME	HE			
Neutrophil, %	75.6	76.7	78.9	1.99	12	0.5013
Lymphocyte, %	23.4	22.0	20.2	1.97	12	0.5223
Cortisol, ng/mL	19.0	22.1	37.6	7.87	12	0.2186
Norepinephrine, ng/mL	25.1	34.6	35.8	5.10	12	0.2795
Glucose, mg/dL	179.9^b^	194.0^ab^	233.6^a^	14.66	12	0.0383
Blood urea nitrogen, mg/dL	19.5	20.5	21.2	0.95	12	0.4377
Creatine, mg/dL	0.6	0.6	0.5	0.05	12	0.7213

^ab^Means with different superscripts differ significantly (*P* < 0.05) by the *P*diff procedure.

LE, low epinephrine group; ME, medium epinephrine group; HE, high epinephrine group.

### Carcass and Meat Quality Characteristics

The ER did not significantly affect live weights, hot and cold carcass weights, and liver weights in goats. Glycogen concentrations measured at 15 min postmortem were significantly influenced (*P* = 0.0011) by ER grouping (Table 2). The concentrations were higher in the ME and LE groups compared to the HE group. *Longissimus lumborum* muscle pH and temperature values measured at 15 min and 24 h postmortem were not significantly affected by treatment. Meat color, WBSF, cooking loss, and desmin levels were also not influenced by ER. However, muscle calpastatin levels tended (*P* = 0.0609) to be higher in the ME group compared to the HE and LE groups (Table 2).

**Table 2.** Effects of differences in epinephrine response to acute stress on body weights and carcass and meat quality characteristics in goats

Variable	Epinephrine response level			SEM	*n*	*P*-value
	LE	ME	HE			
Pre-holding weight, kg	28.3	29.1	27.5	0.90	12	0.4757
Post-holding weight, kg	27.2	27.2	25.8	0.90	12	0.4516
Hot carcass weight, kg	14.4	14.9	13.9	0.74	12	0.6371
Cold carcass weight, kg	11.6	11.5	11.5	0.74	12	0.9927
Liver weight, g	298.4	304.8	241.4	29.42	12	0.2569
Muscle pH 15 min	6.9	6.8	6.9	0.08	12	0.4700
24 h	6.0	6.1	6.1	0.10	12	0.8148
Muscle temperature 15 min	26.5	26.1	25.4	0.47	12	0.2792
24 h	10.5	10.6	10.9	0.29	12	0.5032
Muscle glycogen, µg/µL	8.1^a^	8.1^a^	6.2^b^	0.36	12	0.0011
Color L^*^	39.8	40.3	39.5	0.95	12	0.8579
a^*^	9.7	9.6	8.6	0.48	12	0.2192
b^*^	10.6	10.1	9.6	0.51	12	0.3757
Warner-Bratzler shear force, kg	3.9	3.2	3.1	0.39	12	0.3315
Cooking loss, %	17.3	18.1	15.5	0.94	12	0.1440
Calpastatin, ng/mL	0.8	0.9	0.7	0.04	12	0.0609
Desmin, ng/mL	2.3	2.5	2.1	0.18	12	0.3351

^ab^Means with different superscripts differ significantly (*P *< 0.05) by the *P*diff procedure.

LE, low epinephrine group; ME, medium epinephrine group; HE, high epinephrine group.

### Correlation Analysis

**Figure 1.**
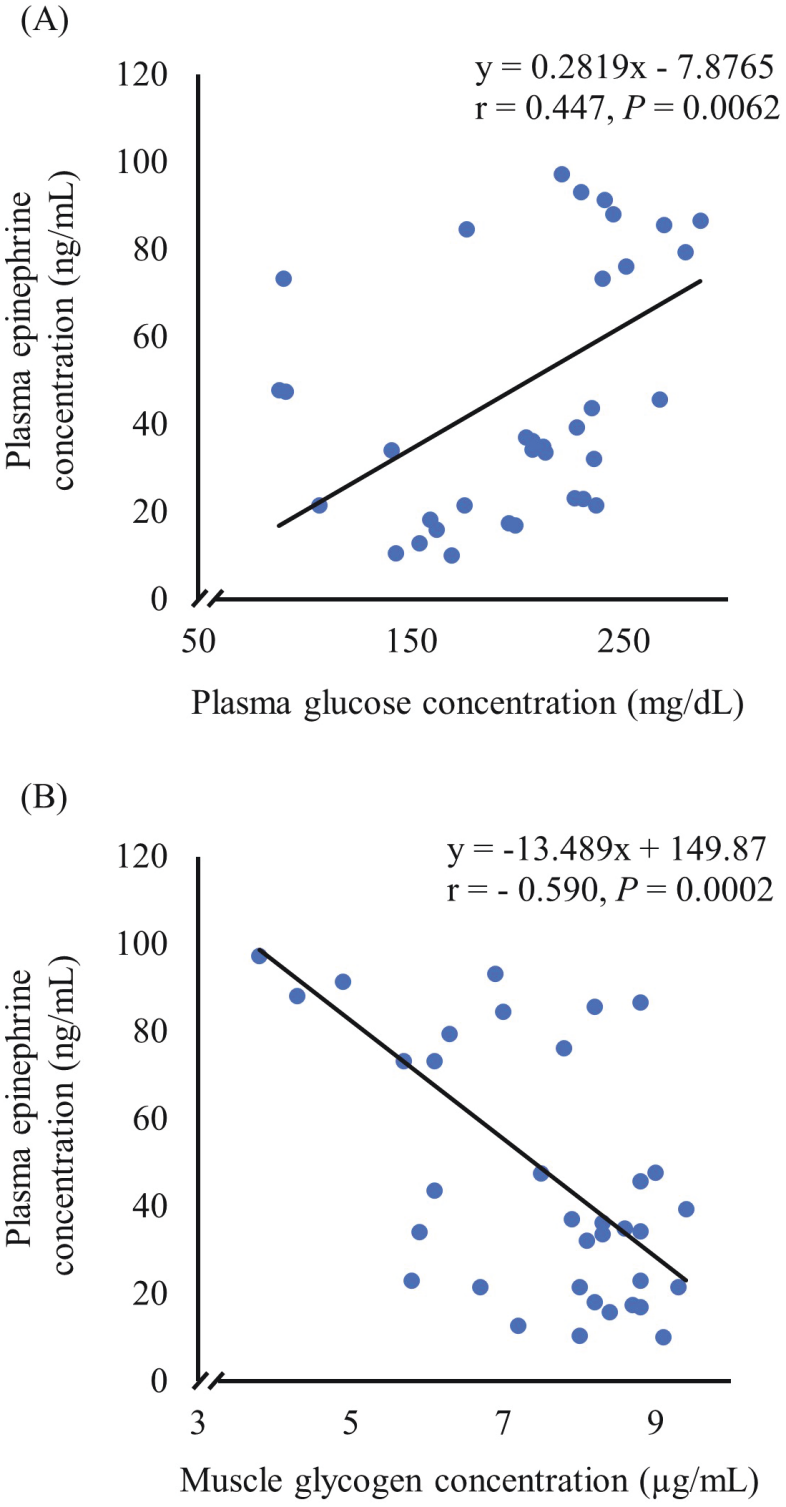
Scatterplots showing the correlation between (A) plasma epinephrine and glucose concentrations, and (B) plasma epinephrine and muscle glycogen concentrations, based on Pearson correlation analysis.

Pearson correlation analysis between epinephrine concentrations and other physiological and meat quality variables measured showed that there were significant correlations between plasma epinephrine and glucose concentrations (*r* = 0.447, *P* = 0.0062, Figure 1A) and between epinephrine and muscle glycogen concentrations (*r* = −0.590, *P* = 0.0002, Figure 1B).

### Muscle Metabolomics

**Figure 2.**
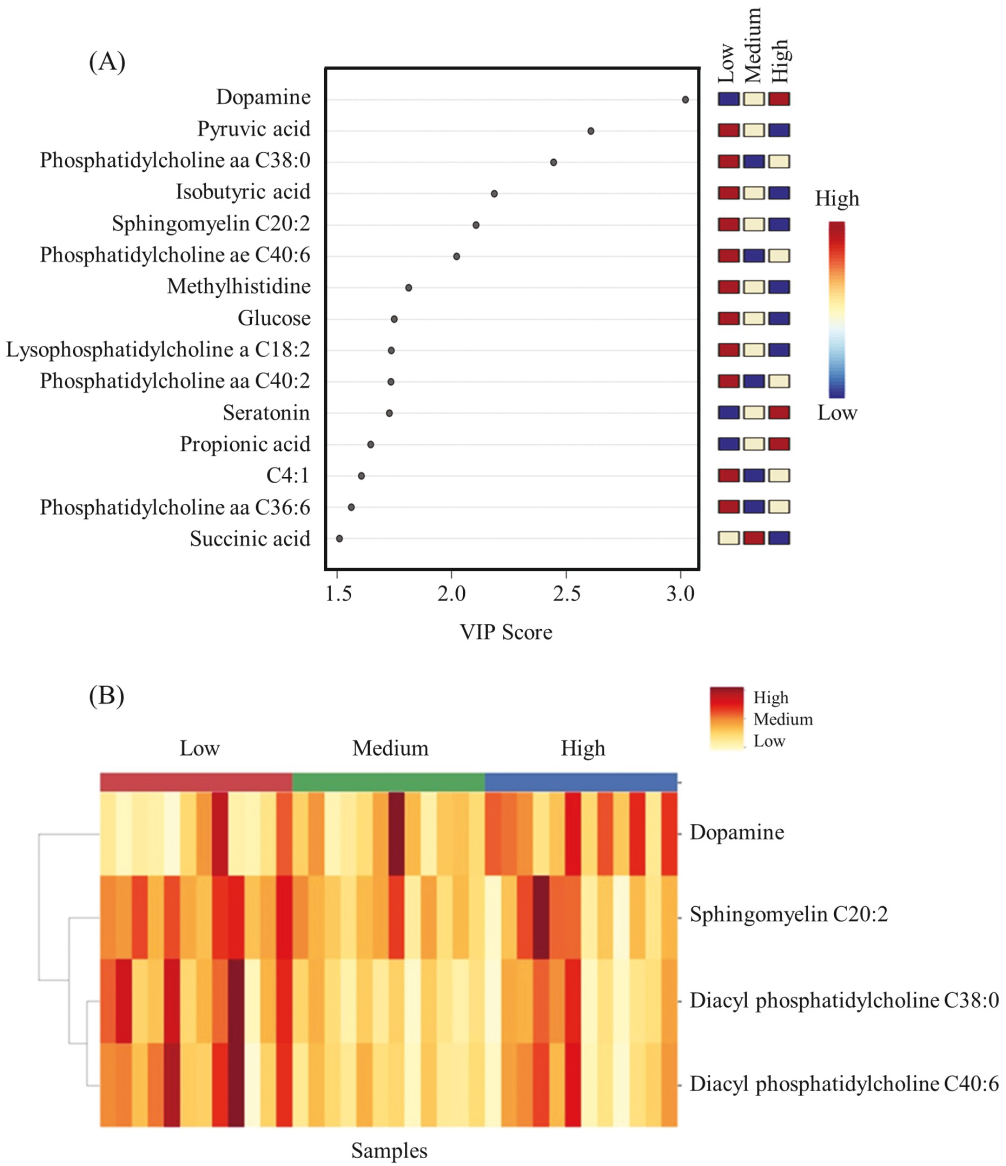
(A). PLS-DA variable importance in projection (VIP) plot showing differences among epinephrine response (ER) groups and the metabolites (VIP scores > 1.5) that significantly contribute to the difference. (B) Heatmap showing the relative abundance of significantly affected (*P* < 0.05) metabolites in the three ER groups.

**Figure 3.**
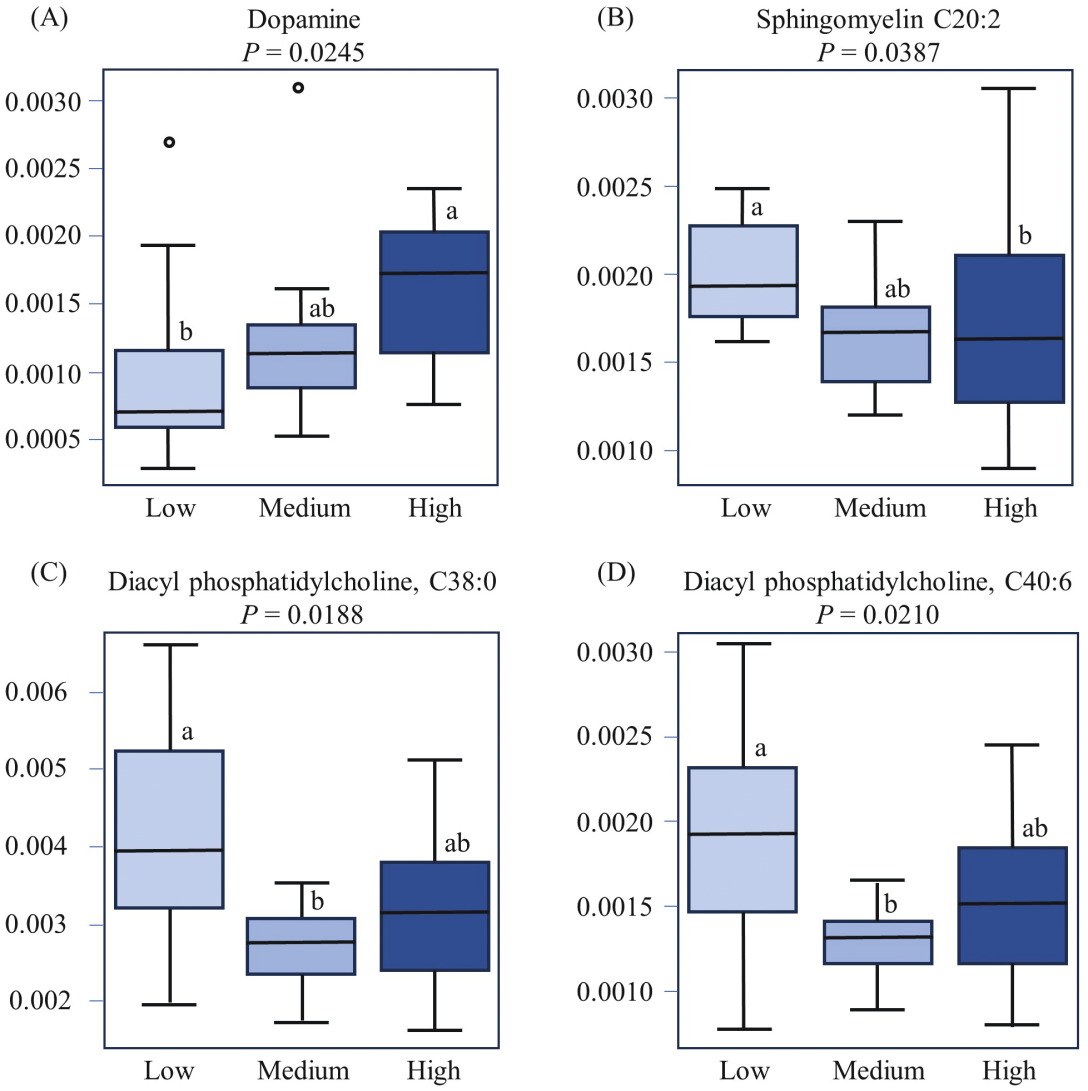
Box plots showing the differences among the three epinephrine response groups in (A) dopamine, (B) sphingomyelin C20:2, (C) diacyl phosphatidylcholine C38:0, and (D) diacyl phosphatidylcholine 40:6 concentrations.

The principal component analysis plot created to visualize the separation of metabolites by ER in principal components 1 and 2 showed that the clusters corresponding to different ER groups overlapped. Dopamine, pyruvic acid, phosphatidylcholine aa C38:0, isobutyric acid, sphingomyelin C20:2, phosphatidylcholine ae C40:6, methylhistidine, glucose, lysophosphatidylcholine a C18:2, phosphatidylcholine aa C40:2, serotonin, propionic acid, C4:1, phosphatidylcholine aa C36:6, and succinic acid were the top 15 metabolites identified by PLS-DA multivariate model (*P* < 0.05) and VIP values with the highest influence (VIP scores > 1.5) in separating the high, medium, and low ER groups (Figure 2A). Non-parametric ANOVA revealed that only four metabolites (diacyl phosphatidylcholine C38:0 [*P* = 0.019], diacyl phosphatidylcholine 40:6 [*P* = 0.021], dopamine [*P* = 0.025], sphingomyelin C20:2 [*P* = 0.039]) were significantly affected by ER. The changes in concentrations of the metabolites that were significantly influenced by ER are shown using a heatmap (Figure 2B). Post hoc pairwise comparisons showed that dopamine concentrations were high in the HE group, low in the LE group, and intermediate in the ME group (Figure 3A). Sphingomyelin C20:2 concentrations were high in the LE group, low in the HE group, and intermediate in the ME group (Figure 3B). Both diacyl phosphatidylcholine C38:0 and diacyl phosphatidylcholine C40:6 concentrations were high in LE, low in ME, and intermediate in the ME group (Figure 3C and D).

## Discussion

### Blood Hormone Concentrations

The present study was conducted under the premise that individual goats differ in their alarm reaction to a stressor and these animal differences could differentially affect postmortem muscle metabolism. Grandin (1980) suggested that epinephrine levels can be sensitive indicators of an animal’s responsiveness to acute stress during events like transportation. Finkemeier et al. (2018) emphasized the significance of investigating and comprehending animal diversity. The authors stated that not only do breeds differ in catecholamine synthesis, but each animal uniquely responds to a particular external stimulus. Curley et al. (2006) and Burdick et al. (2010) reported that the endocrine responses to stress may vary depending on several factors, including the individual animal’s temperament.

Grouping by ER level did not have an effect on the norepinephrine concentrations in the present study. Kadim et al. (2007) found elevated levels of both epinephrine and norepinephrine after transport in small ruminants. However, Nwe et al. (1996) did not observe a concomitant increase in both catecholamines after transportation in goats, likely due to factors such as the species, duration and conditions of transport, and environmental factors such as ambient temperature (Al-Arby et al., 2014). Plasma cortisol concentrations were also not affected by ER levels in the present study. The cortisol response to a stressor may be different from animal to animal as is the ER. Physiological and behavioral adaptations to stress comprise two distinct components (Birhanu, 2020). The initial phase is an “alarm” reaction, characterized by the activation of the SAM axis and the release of catecholamines (Chu et al., 2021). The second component involves the hypothalamic-pituitary-adreno-cortical axis, which triggers the adrenal cortex to release corticosteroid hormones (Birhanu, 2020). Since different neuroendocrine pathways control the two responses, the increase in epinephrine is not always accompanied by an increase in cortisol concentration at a given point in time during or after stress.

### Blood Metabolite Concentrations

Plasma glucose levels are used as indicators of stress in small ruminants (Teke et al., 2014). In the present study, ER grouping significantly affected plasma glucose concentrations, with the levels increasing with increasing ER levels. It is well documented that during stress, catecholamines activate glycogenolysis, resulting in increases in blood glucose and muscle lactic acid levels (Kannan et al., 2003; Romero and Butler, 2007; Adenkola and Ayo, 2010). Transportation stress affects carbohydrate metabolism, as shown by Zulkifli et al. (2010) and Gebresenbet et al. (2012). Individual animal differences in ER also seem to result in differences in glucose concentrations as evidenced by the significant correlation between the two variables since the increase in glucose concentration during stress is a direct effect of an increase in catecholamine concentration. In contrast, no significant differences were observed in plasma BUN and creatine concentrations due to ER levels. Blood urea nitrogen can suggest protein catabolism and its concentration increases during stress (Kouakou et al., 1999); however, it is not the best marker for protein breakdown since other factors such as protein intake can also increase BUN levels (Sahlu et al., 1992). The increase is more related to cortisol release and the resultant increased gluconeogenesis rather than epinephrine release.

### Differential Leukocyte Counts

The differential count of leukocytes, particularly neutrophils, and lymphocytes, is often used for assessing stress in livestock. In our study, ER did not significantly affect leukocyte counts. Endogenous cortisol and catecholamines may be important factors that affect leukocyte counts, particularly neutrophils and lymphocytes (Buonacera et al., 2022). In goats, it is well documented that stress-induced cortisol increase results in increased neutrophil counts and decreased lymphocyte counts (Kannan et al., 2000; Batchu et al., 2021). Stress results in the release of glucocorticoids, which interferes with the immunological response (Marketon and Glaser, 2008) because of a switch from humoral to cell-mediated immunity, thus reducing the immune response (Inbaraj et al., 2016). Epinephrine increase can also cause leukocytosis and lymphopenia (Buonacera et al., 2022). However, there is abundant evidence that stress-induced epinephrine increase results in an acute inflammatory reaction, in contrast to the immune suppressive effects of glucocorticoids, particularly when stress is as a result of or combined with infection or injury (Bergmann and Sautner, 2002; Flierl et al., 2007; Kim et al., 2014).

### Live and Carcass Weights and Meat Quality Characteristics

The reactivity to stress is unique to each animal and it is assumed that this is consistent throughout its life. Individual differences in physiological responses to stress may be influenced by moderator variables, such as personality in humans (Soliemanifar et al., 2018). Finkemeier et al. (2018) stated that such diversities exist not only among breeds but also among individual animals. Considering these viewpoints, a goat can go through numerous stressors during its life and its sympathomedullary arousability level to each stressor is likely to be consistently low, medium, or high throughout. However, it is not known if these variations can influence performance variables such as weight gain and carcass yield. In our study, epinephrine reactivity level did not influence live weights and hot and cold carcass weights.

In the current study, epinephrine reactivity had a significant influence on muscle glycogen concentrations at 15 min postmortem. The HE group had considerably lower glycogen concentrations compared to ME and LE groups. The results of the current study are consistent with the well-established relationship between elevated epinephrine levels and rapid glycogen depletion (Apple et al., 1995; Kadim et al., 2007; Naldurtiker et al., 2022). Epinephrine specifically activates glycogen phosphorylase, the enzyme that breaks down glycogen, while inhibiting glycogen synthase, the enzyme that stimulates glycogen production (Feher, 2017). In the present study, the considerable differences in muscle glycogen concentrations and their association with differences in ER as shown by correlation analysis suggest that animal differences, if substantial, can potentially affect postmortem glycolysis rate. A recent study in goats showed that stress-induced catecholamine increase can deplete glycogen and darken the meat (Naldurtiker et al., 2022). However, *Longissimus dorsi* muscle pH values measured at both 15 min and 24 h postmortem were not influenced by epinephrine reactivity despite noticeable differences in glycogen concentrations. Similar observations were made in earlier studies in goats (Kannan et al., 2003; Naldurtiker et al. 2022) although Mapiye et al. (2011) showed that when epinephrine concentrations increased, ultimate pH values of meat decreased in a linear fashion in steers.

Antemortem depletion of muscle glycogen stores may drastically change meat quality characteristics, including tenderness, aging potential, color, and water-holding capacity (Gregory, 2008). In our experiment, ER had no significant effect on the WBSF, cooking loss, and color values (L*, a*, b*) of rib chops. Meat color is a major factor determining the purchase preference of consumers and this variable along with tenderness, juiciness, and flavor affects the eating quality of meat. The lack of a significant effect of ER on color, WBSF, and cooking loss can be attributed to the fact that ER level, though significantly affecting muscle glycogenolysis, did not influence postmortem muscle pH, which is a major determinant of meat quality development. One of the most important factors in postmortem meat tenderization is the calpain/calpastatin system, which is also pH-dependent (Kannan et al., 2014). A previous study has shown that pre-rigor (15 min postmortem) activities of µ-calpain and m-calpain are 82.1 ± 2.42 and 45.9 ± 1.86 caseinolytic units per 50 g of tissue, respectively, in goat *Longissimus dorsi* muscle (Kouakou et al., 2005). While calpains are responsible for proteolysis, calpastatin inhibits calpain activity and the meat tenderization process. The muscle protein, desmin, a site of endogenous proteolytic activity was also unaffected by ER level in this study. The reason for the trend in our research toward greater calpastatin abundance in the ME group compared to the HE and LE groups is unclear. Mapiye et al. (2011) also did not observe any relationships between epinephrine concentrations and WBSF, water-holding capacity, and cooking loss in beef. It is not known if cold shortening, a common phenomenon in goat carcasses (Kannan et al., 2014), occurred in the current study that could have masked the effects of epinephrine reactivity on meat quality. Hourly temperature and pH values of the *Longissimus lumborum* samples postmortem were not determined in the present study.

### Muscle Metabolomics

The focus of the current investigation was to comprehend the complex relationship between epinephrine reactivity induced by transport and the resulting changes in *Longissimus lumborum* muscle metabolites in goats and their possible relationships to meat quality. Targeted metabolomics analysis revealed that ER significantly affected four metabolites: diacyl phosphatidylcholine C38:0, diacyl phosphatidylcholine C40:6, sphingomyelin C20:2, and dopamine.

Diacyl phosphatidylcholines (C38:0; C40:6) were highest in the LE group, suggesting that goats with low ERs had a differential phospholipid metabolism compared to other groups. Phosphatidylcholines (PC) have a glycerol backbone with two fatty acids that are connected via an ester bond at the sn-1 and sn-2 positions and a polar head (Vance, 2015). Phospholipids and diacyl phosphatidylcholines, which are a form of phospholipid, are required for cell membrane integrity (Mulder et al., 2003; Vance and Vance, 2008). These metabolites are essential for neuronal and synaptic structure as they play a crucial role in the signal transduction responses to neurotransmitters (Horrobin, 2001). Phosphatidylcholines play a crucial role during stress by contributing to endoplasmic reticulum membrane stability and ensuring the cell can respond effectively to stress-induced challenges and maintain cellular homeostasis (Thorne et al., 2010). Lower ER during stress may have several benefits in goats such as better cell integrity, signal transduction, and overall ability to handle stress.

The balance of different types of phosphatidylcholine species has been reported to influence the nutritional quality of pork (Enomoto et al., 2020). The authors observed that diacyl phosphatidylcholines contain polyunsaturated fatty acids such as 18:2 and 20:4 at the sn-2 position. In addition, phosphatidylcholine species contain the bulk of choline in meat (82.6% ± 5.5% total choline), which has several beneficial effects when administered in rodents, including lowering stress and improving memory and cognitive functions (Lewis et al., 2015; Bekdash, 2019). Poutzalis et al. (2018) observed that phosphatidylcholines from goat and sheep meat had anti-inflammatory activity in vitro and potential antithrombotic and cardioprotective properties. Goat meat has higher proportions of polyunsaturated fats compared to other traditional red meats consumed (Park and Washington, 1993), and the elevated concentrations of certain diacyl phosphatidylcholines further support its nutritional benefits. However, these metabolites appear to have no relationship to the eating quality characteristics, such as texture, color, and juiciness of meat. The potential relationship between lower ER to stress in goats and fatty acid profiles of meat needs further investigation.

Sphingomyelin is a major sphingolipid in animal cell membranes and its metabolism generates a variety of bioactive compounds, including ceramides, sphingosine, and sphingosine-1-phosphate. These molecules have major functions in cellular signaling and stress responses (Hannun and Obeid, 2018). The increase in stress hormones seems to interfere with peripheral sphingolipid metabolism (Miller et al., 2016). The elevated levels of sphingomyelin detected in the LE group in our study indicate that it may have a protective or compensatory function during acute stress. Sphingomyelin may be mobilized by cellular mechanisms due to low ER during stress to maintain the integrity of cell membranes and provide protection against potential cellular injury. The reduced levels of sphingomyelin in the HE groups could indicate an augmented breakdown of membrane lipids to supply immediate energy or precursors for other metabolites required during a stress response. Lower epinephrine reactivity and elevated sphingomyelin levels could confer a protective or adaptive effect in muscle tissue during transport stress; however, its implications for meat quality are not evident based on the variables assessed in this study.

Dopamine and epinephrine, both essential catecholamines in the neurological and endocrine systems, have a complex metabolic interaction. Dopamine is a prominent neurotransmitter that plays a crucial role in mood, behavior, and stress response (Belujon and Grace, 2017). The present study showed that ER levels in goats significantly affected dopamine concentrations, and this effect was not unexpected given that dopamine is a precursor of norepinephrine and epinephrine. Previous studies have demonstrated that acute stresses may increase dopamine release (Kadim et al., 2006; Valenti et al., 2011), and proper dopamine regulation is vital for a balanced stress response (Illiano et al., 2020). Simultaneous increases in both epinephrine and dopamine concentrations in response to acute stress in livestock have been previously reported. For example, Kadim et al. (2006) observed that stress caused higher norepinephrine, epinephrine, and dopamine levels in Omani goats compared to non-stressed counterparts.

Muscle metabolomic profiles did not change much in response to epinephrine reactivity in goats, except for a few metabolites. These metabolites were not related to any of the meat quality characteristics assessed in this study, however.

The limitation of this study is that goat carcasses are prone to cold shortening due to the smaller size and lack of fat coverage which allows rapid heat dissipation during the initial hours in the cooler yielding less tender meat. If cold shortening had occurred in the carcasses in the present study, it could have overridden the differences in meat quality caused by epinephrine reactivity.

## Conclusions

Animal differences in epinephrine reactivity to stress did not result in alterations in postmortem muscle pH and meat quality characteristics, although postmortem muscle glycogen concentrations were affected by and correlated with ER. Lower ER level to stress is likely beneficial to goats as shown by higher concentrations of diacyl phosphatidylcholines and sphingomyelins in this group. Overall, animal differences in ER level did not significantly affect meat quality characteristics. Further research is required to better understand the relationships among animal personality, stress reactivity, and meat quality in goats.

## Supplementary Material

txae078_suppl_Supplementary_Figure
