# Supplementary material for: Influence of epinephrine reactivity to stress on meat quality in goats
Source: Transl Anim Sci. 2024 May 28;8:txae078. doi: 10.1093/tas/txae078 (PMC11143493; doi:10.1093/tas/txae078)
Supplement: txae078_suppl_Supplementary_Figure [file txae078_suppl_supplementary_figure.pptx]

## Slide 1
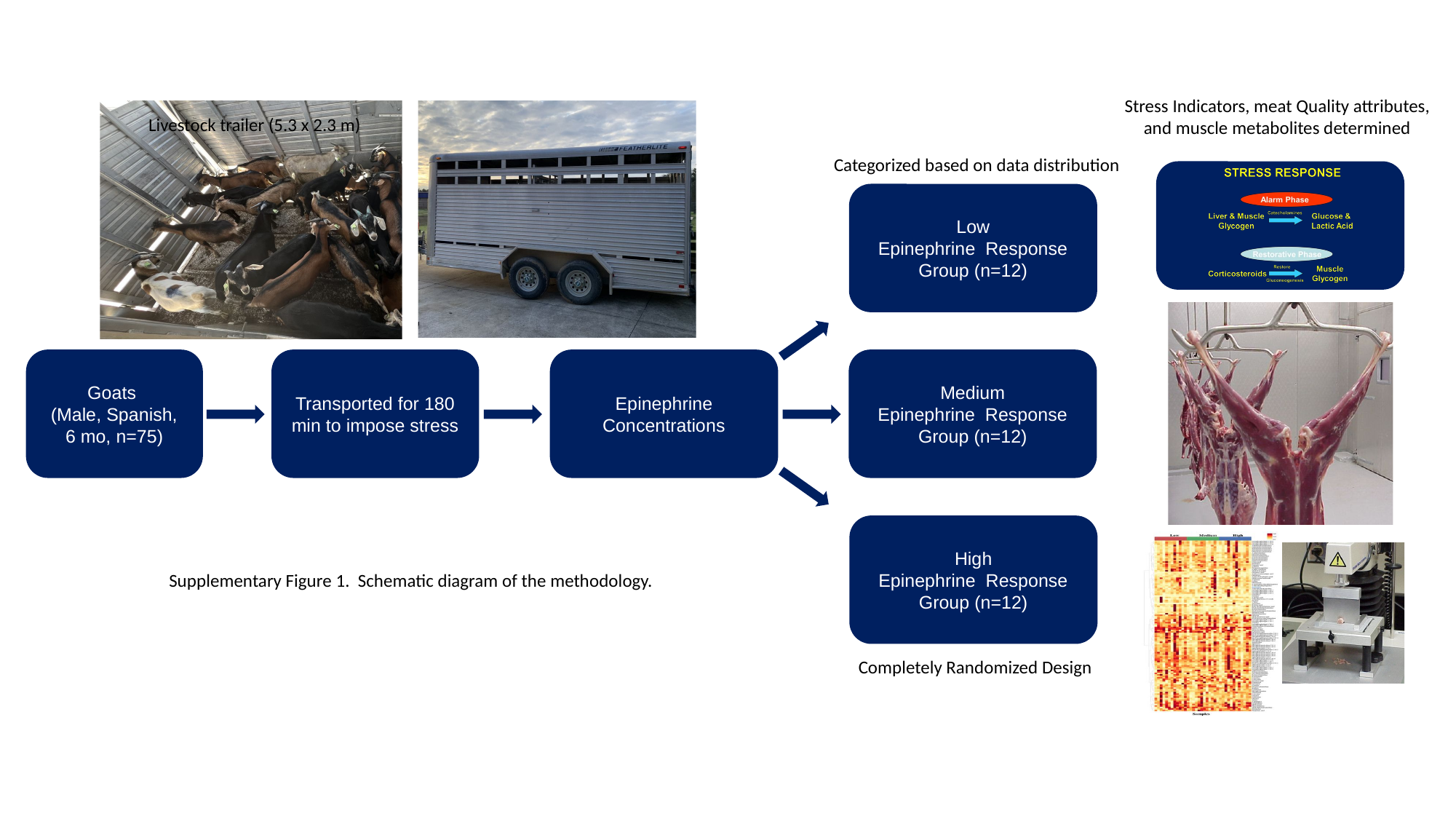

Stress Indicators, meat Quality attributes,
and muscle metabolites determined
Livestock trailer (5.3 x 2.3 m)
Categorized based on data distribution
Low
Epinephrine Response Group (n=12)
Goats
(Male, Spanish,
6 mo, n=75)
Transported for 180 min to impose stress
Epinephrine
Concentrations
Medium
Epinephrine Response Group (n=12)
High
Epinephrine Response Group (n=12)
Supplementary Figure 1. Schematic diagram of the methodology.
Completely Randomized Design
